# Post-Hemiplegic Athetosis

**Published:** 1907-11-02

**Authors:** 


### Illustrative Cases.

### POST-HEMIPLEGIC ATHETOSIS.

Athetosis following hemiplegia, sometimes misnamed post-hemiplegic chorea, is by no means uncommon, though but a small proportion of all hemiplegia cases exhibit the phenomenon. The reason why it is not more common is that athetosis is the result of cortical irritation, and comparatively few cases of hemiplegia are due to a cortical lesion; the majority are the result of subcortical trouble, hæmorrhage near or into the internal capsule, the cortex itself remaining healthy. The following is an example of a cortical hæmorrhage followed by athetosis; that the lesion was cortical and not subcortical is indicated not only by the athetosis, but also by the typical motor aphasia which was present at the beginning.

Agnes G., aged 57, had been for some time addicted to alcohol, and one evening, soon after returning home, she was seized with giddiness, soon after which she fell forward on to the floor without losing consciousness. Her husband, on going to raise her up, found her unable to use her left arm or left leg, and he noticed that her mouth was twisted. The power of speech was also lost. The facial paralysis affected only the parts below the eye. There was no paralysis of the tongue. Although speech was lost, the patient understood what was said to her perfectly, she could read and understand things written, though she could not write owing to paralysis of her right hand; she understood the uses of different objects shown to her; in other words, she had typical motor aphasia and left-sided hemiplegia.

The aphasia lasted about a month, and then slowly but surely improved until complete power of speaking was recovered. The facial palsy also disappeared.

The paralysis of the right leg began to get better long before that of the right arm showed any signs of improvement; the leg did not become absolutely well, but the patient was able to get about with a limping gait long before the arm could be used for anything. Later, however, power began to return to the arm in the usual order—the shoulder being moved voluntarily before the elbow, the latter before the wrist, and the latter before the fingers.

About a year after the apoplectic seizure, the patient began to exhibit abnormal movements in her right forearm. The hand, closed in upon the palm, was affected by rhythmical and rapid oscillations which were communicated to the forearm, and at first interfered greatly with the patient's power of holding things. A glass or cup raised by this hand was forthwith dashed to the ground. The movements were absolutely beyond voluntary control, though voluntary movements could be superposed upon the rhythmical athetosis; during sleep the arm and hand were quite quiescent, but the whole time the patient was awake these athetotic movements were incessant. There were no similar trouble in the corresponding leg; the only point to note about the latter was that the great toe assumed a permanent state of extreme dorsiflexion—a sort of perpetual Babinski's sign—so that holes had to be cut in shoes and boots to accommodate it.

Intellectually, the patient was bright and alert, and as time went on she learned to allow for the perpetual coarse tremor of her right hand, accommodating herself to such an extent that she could use a knife, fork, or spoon efficiently, and could even carry a glass of water, fairly full, up to her lips and drink it successfully though the rhythmical involuntary movements were going on all the time. Cold, excitement, or fatigue, each caused an increase in the athetosis, so that the patient never went out of doors without a glove to keep her hand warm, and she avoided undue excitement or physical fatigue as far as possible.

Nothing in the way of curative treatment has proved successful, and it seems likely that the movements will persist just as they are, neither increasing or diminishing, unless a second hæmorrhage should occur in the arm area of the left Rolandic cortex and entirely paralyse the limb.

